# Dual-Delivery Temperature-Sensitive Hydrogel with Antimicrobial and Anti-Inflammatory Brevilin A and Nitric Oxide for Wound Healing in Bacterial Infection

**DOI:** 10.3390/gels10040219

**Published:** 2024-03-24

**Authors:** Linghui Ruan, Chengfeng Pan, Xianting Ran, Yonglan Wen, Rui Lang, Mei Peng, Jiafu Cao, Juan Yang

**Affiliations:** 1 State Key Laboratory of Functions and Applications of Medicinal Plants, Guizhou Medical University, Guiyang 550014, China; ruanlinghui1997@163.com (L.R.); pengmei520@163.com (M.P.); 2 Natural Products Research Center of Guizhou Province, Guiyang 550014, China; panchengfeng0422@163.com (C.P.); ranxianting@163.com (X.R.); wyonglan0217@163.com (Y.W.); lr1792077492@163.com (R.L.)

**Keywords:** brevilin A, Pluronic F127, S-nitrosoglutathione, antibacterial, anti-inflammatory, wound healing, hydrogel

## Abstract

Bacterial infections impede the wound healing process and can trigger local or systemic inflammatory responses. Therefore, there is an urgent need to develop a dressing with antimicrobial and anti-inflammatory properties to promote the healing of infected wounds. In this study, BA/COs/NO-PL/AL hydrogels were obtained by adding brevilin A (BA) camellia oil (CO) submicron emulsion and nitric oxide (NO) to hydrogels consisting of sodium alginate (AL) and Pluronic F127 (PL). The hydrogels were characterized through dynamic viscosity analysis, differential scanning calorimetry, and rheology. They were evaluated through anti-inflammatory, antimicrobial, and wound healing property analyses. The results showed that BA/COs/NO-PL/AL hydrogels were thermo-responsive and had good ex vivo and in vivo anti-inflammatory activity, and they also exhibited strong antimicrobial activity against methicillin-resistant Staphylococcus aureus *Pseudomonas aeruginosa* (MRPA) and methicillin-resistant *Staphylococcus aureus* (MRSA). They were able to effectively promote healing of the infected wound model and reduce inflammation and bacterial burden. H&E and Masson’s staining showed that BA/COs/NO-PL/AL hydrogels promoted normal epithelial formation and collagen deposition. In conclusion, BA/COs/NO-PL/AL hydrogels are promising candidates for promoting the healing of infected wounds.

## 1. Introduction

The skin, being the biggest organ of the human body, serves as the primary barrier against many environmental stimuli [1,2,3]. When an injury occurs, such as from trauma, surgery, or burns, the skin’s fundamental tissue structure and essential functions are disrupted, resulting in the formation of a wound. Inadequate attention to the wound healing process might result in infection caused by bacteria and other infectious agents [4,5]. Pathogenic microorganisms quickly establish themselves in the wound and release substantial quantities of exudate, impeding the progression from wound healing to later stages of cell growth and tissue remodeling. This delay in wound healing can potentially heighten the chances of amputation and mortality [6,7].

Antibiotics are currently an efficacious method for treating and preventing infected wounds; however, their improper application renders bacteria vulnerable to drug resistance and potentially gives rise to superbugs [8]. Hence, the investigation and advancement of novel antimicrobial agents to manage infected wounds are notably valuable and consequential. As a novel antimicrobial agent, nitric oxide (NO) is the subject of extensive research due to its capacity to disperse and destroy bacterial biofilms directly, thereby preventing the emergence of drug resistance [9,10]. This is because the reaction of NO with oxygen generates reactive nitrogen oxide species (RNOS) spontaneously, which induces lipid peroxidation and oxidation in bacterial cells via DNA modification, enzyme inhibition, and nitrosative damage [11]. Substances that have been nitrosylated disrupt the functionality of critical proteins and cause bacterial cell death. Peroxynitrite (ONOO^−^) is generated when NO reacts with endogenous superoxide generated during bacterial respiration. This compound induces oxidative damage to lipids and DNA [9]. NO functions as a signaling molecule and an antimicrobial agent, in addition to regulating various organs and tissues [12]. This includes the epidermis, where it affects wound healing, osmotic barrier homeostasis, and oxidative stress. Furthermore, NO exerts anti-inflammatory effects on the skin and extracutaneous tissues. NO, however, is unsuitable for wound healing due to its gaseous state and short half-life. To maximize the antimicrobial properties of NO, numerous NO donors, including azodiolenes, nitrosothiols, nitrobenzenes, metal nitrosyl compounds, organic nitrates, and organic nitrites, have been developed to deliver NO continuously [13].

Over the very first stage of investigation, our group formulated a thermo-responsive hydrogel (GSNO-PL/AL) consisting of alginate (AL), Pluronic F127 (PL), and S-nitroso glutathione (GSNO). This hydrogel exhibits robust bactericidal activity against multidrug-resistant *Pseudomonas aeruginosa* (MRPA) and methicillin-resistant *Staphylococcus aureus* (MRSA). Furthermore, it expedites the healing process of MRPA-infected wounds and diminishes the bacterial load on the affected area. Nevertheless, during the initial phase, the investigation of GSNO-PL/AL hydrogel was limited to its antibacterial properties; its potential anti-inflammatory effects on the healing process of infected wounds were not assessed. In addition to bacterial infection, inflammation is also a factor in the delayed healing of infectious wounds [14]. Inflammation ensues due to bacterial invasion of the lesion. Tissue edema, ulceration, and necrosis, which ensue from an exaggerated inflammatory response, severely impede the progression from wound healing to subsequent proliferation and remodeling. This can lead to chronic wound healing or even complete non-healing [15,16,17,18,19].

Brevilin A (BA) possesses anti-inflammatory properties [20,21], acts as an antioxidant [22,23], enhances skin inflammation [24], and exhibits various other biological functions. Although BA exhibits remarkable anti-inflammatory properties, it struggles with limited water solubility and low bioavailability. In recent years, researchers have explored different types of nanocarriers, including polymeric nanoparticles, lipid nanocarriers, and surfactant nanocarriers (such as nano-emulsions, micro-emulsions, and submicron emulsion) to enhance the delivery of poorly water-soluble drugs for topical skin therapy. These nanocarriers have shown promise in improving drug delivery in dermatoses that damage the skin barrier [25]. Submicron emulsion as a new type of drug carrier system has the advantages of improving drug solubility and stability, preventing drug oxidation, improving in vivo and percutaneous absorption of drugs, and making drugs slow-release, controlled-release, targeted, etc. It has been used in the study of various routes of drug delivery, such as injections and transdermal, oral, and mucosal drug delivery [26,27]. Fernandez et al. [28] prepared a submicron emulsion of benzophenone-3 and compared it with five other carriers, such as crude milk, in transdermal experiments, which showed that the best transdermal effect was achieved with submicron emulsion as the carrier, which was three times higher than that of the crude milk, and the retention in the stratum corneum was the highest. The use of sub-microemulsion as a drug carrier and the combination of gel technology to construct sub-microemulsion gels with high drug loading capacity and good slow and controlled release has a good development prospect [29].

Camellia oil (CO), an edible oil of superior quality, is derived from the seeds of Camellia oleifera Abel. Notably, it exhibits potential as a drug carrier [30]. Burns are frequently treated with CO in traditional medicine, and research has demonstrated that CO possesses antioxidant, protective, anti-inflammatory, and antibacterial properties [24,31,32]. Hence, in this investigation, CO was employed as the submicron emulsion oil phase due to its capacity to facilitate BA solubility increase and wound healing. Consequently, BA submicron emulsion (BA/COs) was formulated using CO.

In pursuit of more effective therapeutic agents, GSNO-PL/AL hydrogels were enhanced in this study. In order to rationally design a hydrogel dressing that possesses both antimicrobial and anti-inflammatory properties, BA/COs (anti-inflammatory) was incorporated into GSNO-PL/AL hydrogel (antimicrobial), resulting in the formation of BA/COs/NO-PL/AL hydrogel. By eliminating inflammation and inhibiting wound infection, it enhances the healing process of infected wounds.

Herein, in order to evaluate the effectiveness of BA/COs/NO-PL/AL hydrogels in healing infected wounds, the hydrogels were analyzed using gel temperature, differential scanning calorimetry (DSC), and in vitro release tests. The objective is to investigate the in vitro antimicrobial activity of BA/COs/NO-PL/AL hydrogels against MRPA and MRSA, as well as their anti-inflammatory activity against RAW264.7 cells and cytotoxicity against L929 cells. Ultimately, the therapeutic effect of BA/COs/NO-PL/AL was assessed in a mouse wound model infected with MRPA.

## 2. Results and Discussion

### 2.1. Characterization of Submicron Emulsion

A uniform and stable milky white emulsion of COs and BA/COs was achieved using ultrasound-assisted homogenization. Following centrifugation at a speed of 4000 rpm/min for a duration of 15 min, no separation occurred, no oil droplets were formed, and the Ke values were minimal, suggesting that both COs and BA/COs exhibited stable features.

The particle size distribution coefficient and zeta potential of the formulations were determined using a Malvern particle size meter. The results (Table 1) indicated that the emulsions had a single peak in the particle size distribution. The droplet sizes of COs and BA/COs were measured to be 237.82 ± 0.54 nm and 239.18 ± 1.47 nm, respectively. The polydispersity indices (PDI) were found to be 0.183 ± 0.04 for both formulations. The zeta potential was −38.67 ± 0.19 mV and −38.25 ± 0.27 mV, respectively. The surface potential of emulsion droplets plays a crucial role in the stability of emulsions, and a higher absolute value of zeta potential indicates that their surfaces are more charged and they will tend to repel each other, thus achieving the stability of the whole system [33]. These findings suggest that the prepared formulations were stable, and the addition of BA did not affect the submicron emulsion formulations.

### 2.2. BA/COs/NO-PL/AL Synthesis and Characterization of Hydrogels

**Figure 1:**
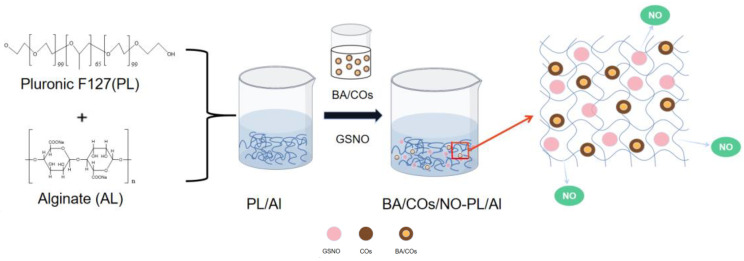
Schematic diagram of PL/AL hydrogel formation containing both NO donor (GSNO) and BA submicron emulsion (BA/COs).

In order to develop a temperature-sensitive hydrogel that can be used as a topical antimicrobial and anti-inflammatory agent, BA/COs/NO-PL/AL hydrogels were synthesized in this study (Figure 1). First, PL was added to the AL solution at 4 °C and dissolved to obtain PL/AL gel. Then, GSNO and BA/COs were added to the PL/AL gel to form the BA/COs/NO-PL/AL hydrogel.

**Figure 2:**
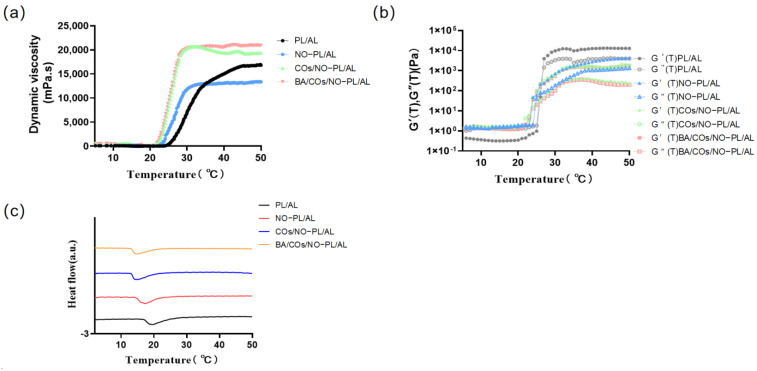
(**a**) Dynamic viscosity studies of PL/AL, NO-PL/AL, COs/NO-PL/AL, and BA/COs/NO-PL/AL hydrogels. (**b**) Temperature rise test of hydrogel. Variation of G′ and G″ with temperature. (**c**) DSC pyrometer for hydrogels.

The gelation temperature of the hydrogels was determined through viscosity studies (Figure 2a, Table 2) describing the temperature dependence of the transition from the sol phase to the gel phase. The dynamic viscosity (Pa.s) values of PL/AL, NO-PL/AL, COs/NO-PL/AL, and BA/COs/NO-PL/AL varied gradually with increasing temperature in the range of 5–50 °C, and from the inflection point, the sol–gel transition presented at 24.5, 22.9, 18.2, and 18.4, respectively. The viscosity trends of COs/NO-PL/AL and BA/COs/NO-PL/AL were the same, indicating that the addition of BA did not affect the gel viscosity. In addition, COs/NO-PL/AL and BA/COs/NO-PL/AL hydrogels had higher dynamic viscosities at the gel point, indicating better resistance to deformation under stress.

The rheometer was used to investigate the variation in viscoelastic characteristics of PL/AL, NO-PL/AL, COs/NO-PL/AL, and BA/COs/NO-PL/AL gels with temperature (Figure 2b). The storage modulus (G′) and loss modulus (G″) of gels are displayed for temperatures ranging from 5 to 50 °C. During the early phase of temperature rise, the values of both G′ and G″ for this system are quite low, with G′ being less than G″. This suggests that the gels are completely dissolved at low temperatures. As the temperature rises, the rate of growth of G′ surpasses that of G″. The temperature at which G′ equals G″ is referred to as the sol–gel phase transition temperature (T_sol-gel_). This signifies the transformation of the hydrogel from a sol state to a gel state. The plots clearly demonstrate that the T_sol-gel_ values for the PL/AL, NO-PL/AL, COs/NO-PL/AL, and BA/COs/NO-PL/AL gels are around 22–24 °C. The hydrogels NO-PL/AL, COs/NO-PL/AL, and BA/COs/NO-PL/AL exhibited a greater T_sol-gel_ compared to PL/AL gel. This increase can be attributed to the incorporation of GSNO and COs or BA/COs, which resulted in a reduction in the PL content. The viscoelasticity trends of COs/NO-PL/AL and BA/COs/NO-PL/AL were identical, suggesting that the inclusion of BA had no impact on the gel’s viscoelastic properties. The G′ and G″ values of COs/NO-PL/AL and BA/COs/NO-PL/AL gels in the gel state were much greater than those of NO-PL/AL gels, suggesting that the incorporation of COs or BA/COs in NO-PL/AL hydrogels resulted in enhanced mechanical strength compared to NO-PL/AL gels.

Once the temperature reaches an appropriate level, F127 monomolecular chains have the ability to come together in a solution and form micelles. This process may be detected through DSC. Hence, the micellization behavior of the hydrogels was assessed by employing DSC mapping, with the ambient temperature serving as the independent variable (Figure 2c, Table 2). The micellization process is defined by the parameters T_onset_, T_peak_, T_endset_, and ΔT. T_onset_ refers to the temperature at which the formation of micellization begins, whereas T_endset_ is the temperature at which the process of micellization is fully finished. The data (s 2) demonstrate that the inclusion of GSNOs, COs, or BA/COs expedited the process of micellization for PL/AL.

### 2.3. BA/COs/NO-PL/AL Drug Dissolution

**Figure 3:**
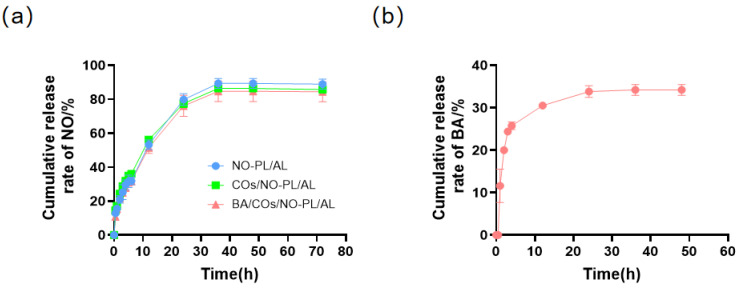
(**a**) Diffusion rates of NO in NO-PL/AL, COs/NO-PL/AL, and BA/COs/NO-PL/AL hydrogels. (**b**) Diffusion rate of BA in BA/COs/NO-PL/AL hydrogels. Data are expressed as mean ± SD (n = 3).

This study investigated the percentage of NO and BA released over time from NO-PL/AL, COs/NO-PL/AL, and BA/COs/NO-PL/AL gels using the Franz diffusion method, as depicted in Figure 3. This suggests that the presence of COs and BA/COs did not have an impact on the release pattern of NO from the hydrogels. Within the initial 5 h of observation, there was a burst release of NO, which was then followed by a consistent release that eventually stabilized after 36 h. The cumulative release rate exceeded 80%, indicating a near-total release of the medication. The diffusion curve of BA in the BA/COs/NO-PL/AL system, as shown in Figure 3b, indicates that BA was not identified at 0.5 h. However, it was released rapidly during the first 3 h, with a cumulative release rate of 24.4%. Subsequently, the release rate slowed down but continued until 24 h, resulting in a cumulative release rate of over 35%. During the initial phase of hydrogel testing, a burst release of BA was observed, which was most likely due to the presence of the drug not encapsulated into the nano-emulsion. Therefore, these findings indicate that PL/AL hydrogels have the ability to facilitate the continuous dispersion of NO and BA.

### 2.4. Cytotoxic Activity of Hydrogels against L929

**Figure 4:**
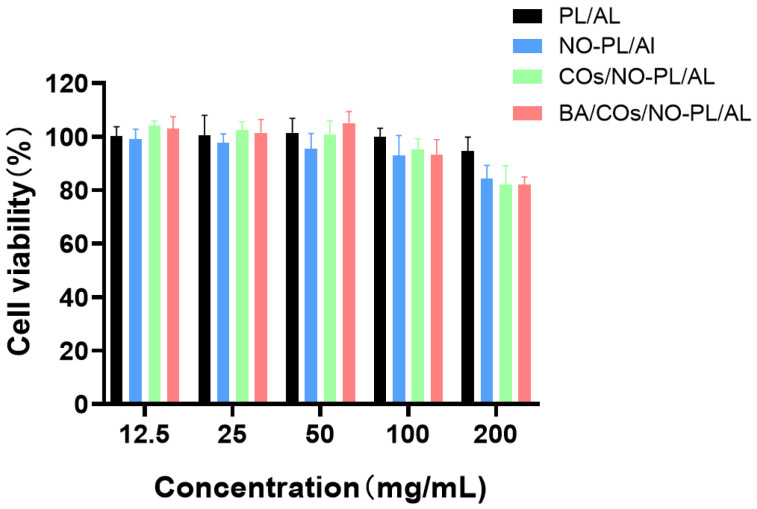
In vitro cytotoxic activity of L929 fibroblasts after incubation with different concentrations of hydrogels for 24 h. Data are expressed as mean ± SD (n = 5).

The effect of NO-PL/AL, COs/NO-PL/AL, and BA/COs/NO-PL/AL gels, with concentrations of 12.5, 25, 50, 100, and 200 mg/mL, on the survival of fibroblasts in L929 mice was assessed using the MTT test. Figure 4 demonstrates that PL/AL hydrogels exhibited negligible cytotoxicity, with cell survival exceeding 90%, owing to the exceptional biocompatibility of PL and AL. The cell viability of NO-PL/AL, COs/NO-PL/AL, and BA/COs/NO-PL/AL was 84.21 ± 4.57%, 82.64 ± 5.80%, and 82.25 ± 2.5%, respectively, at a concentration of 200 mg/mL. The low cytotoxicity of NO-PL/AL, COs/NO-PL/AL, and BA/COs/NO-PL/AL indicated that NO-PL/AL, COs/NO-PL/AL, and BA/COs/NO-PL/AL did not have significant cytotoxic effects on L929 cells. Furthermore, the cell viability decreased with increasing concentration. It has been shown that GSNO is cytotoxic to L929 cells [34]. Previous studies have shown that the inhibitory effect of GSNO on the cytotoxicity of NO-PL/AL is due to the controlled release of NO, which protects the cells from being exposed to a large amount of NO, implying that the NO released from NO-PL/AL can be safely used for topical application [35]. The results showed that NO-PL/AL, COs/NO-PL/AL and BA/COs/NO-PL/AL composite hydrogels were not cytotoxic.

### 2.5. In Vitro Anti-Inflammatory Activity

**Figure 5:**
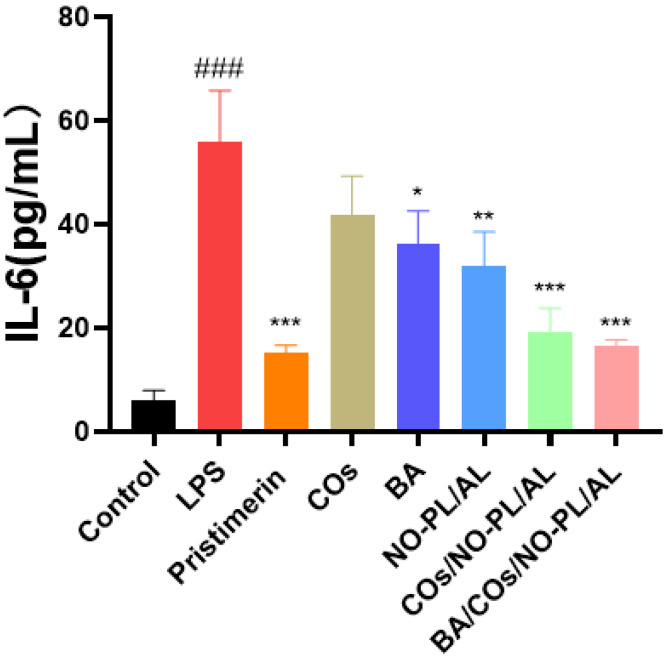
In vitro anti-inflammatory activity of RAW264.7 cells after incubation with different concentrations of hydrogels for 24 h. Data are expressed as mean ± SD (n = 3). ^###^ *p* < 0.001 vs. control. * *p* < 0.05, ** *p* < 0.01, *** *p* < 0.01 vs. LPS.

Inflammation is a basic pathological process and the first sign of a lot of illnesses. Inflammation that lasts for a short time helps the body get rid of inflammatory substances and heal, but inflammation that lasts for a long time puts more stress on the body and damages tissues and organs [36]. IL-6 and other inflammatory agents and cytokines are made by activated macrophages, which are an important part of the body’s defense against infection [37]. Next, we looked into how different samples affected the production of IL-6. Figure 5 shows that the model group that only received LPS increased the production of IL-6 by a very large amount compared to the control group. When LPS was added to macrophages, 200 mg/mL COs had a weak limiting effect on IL-6. Furthermore, 200 mg of NO-PL/AL/mL IL-6 production by macrophages activated by LPS was weakly blocked at 2 µM, 200 mg/mL of COs/NO-PL/AL, and 200 mg/mL of BA/COs/NO-PL/AL. At the amounts tested, COs/NO-PL/AL and BA/COs/NO-PL/AL were better at stopping IL-6 from being made by macrophages that were activated by LPS. Based on the above results, it seems that BA, COs, and NO work together to reduce inflammation, making them more effective.

### 2.6. In Vitro Antibacterial Activity

**Figure 6:**
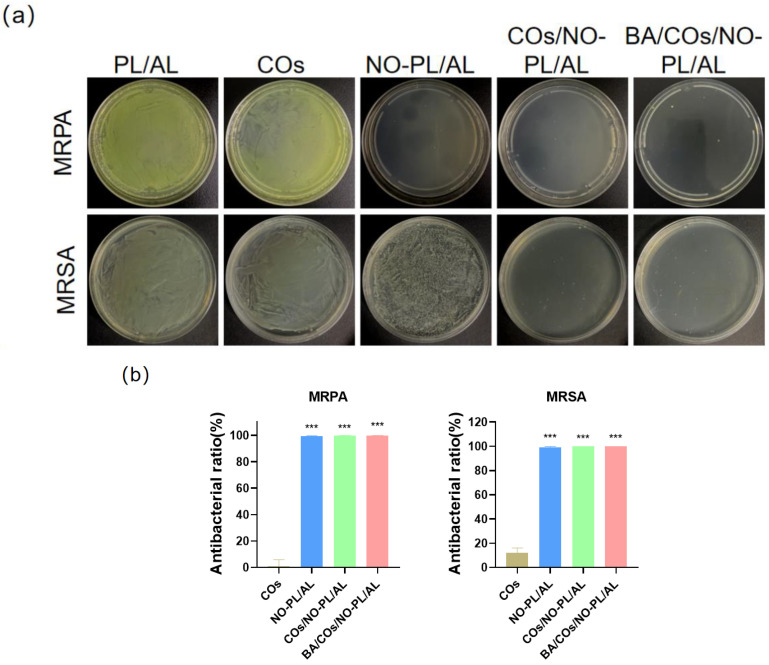
(**a**) Hydrogel colony counting of MRPA and MRSA. (**b**) Quantitative analysis of antimicrobial rate. Data are expressed as mean ± SD (n = 3). *** *p* < 0.01 vs. PL/AL.

To further investigate the antimicrobial properties of BA/COs/NO-PL/AL hydrogels, the in vitro antimicrobial activity of the hydrogels was tested against Gram-negative bacteria (MRPA) and Gram-positive bacteria (MRSA), which are representative pathogens of skin infections. Figure 6 shows the results of in vitro antimicrobial activity following co-incubation with COs, NO-PL/AL, COs/NO-PL/AL, and BA/COs/NO-PL/AL gels at 37 °C for 24 h. COs have little or no inhibitory effect on MRPA and MRSA. This suggests that the antimicrobial activity was generated from NO. Adding COs to NO-PL/AL increased MRSA CFU from 99.09 ± 0.65% to 99.99 ± 0.01% while increasing antibacterial activity. Possible reasons include saponins, phenols, and organic acids in CO, all of which have antibacterial properties against *Staphylococcus aureus*. CO mainly inhibits bacterial growth by influencing the permeability of cell walls and membranes, and it can even impair the integrity of cell membranes [36]. When the permeability of bacterial cell walls and cell membranes is increased or disrupted, so does the permeability of GSNO, resulting in greater antibacterial activity and a synergistic antibacterial effect.

### 2.7. Healing Effect of Hydrogel on MRPA-Infected Wounds

**Figure 7:**
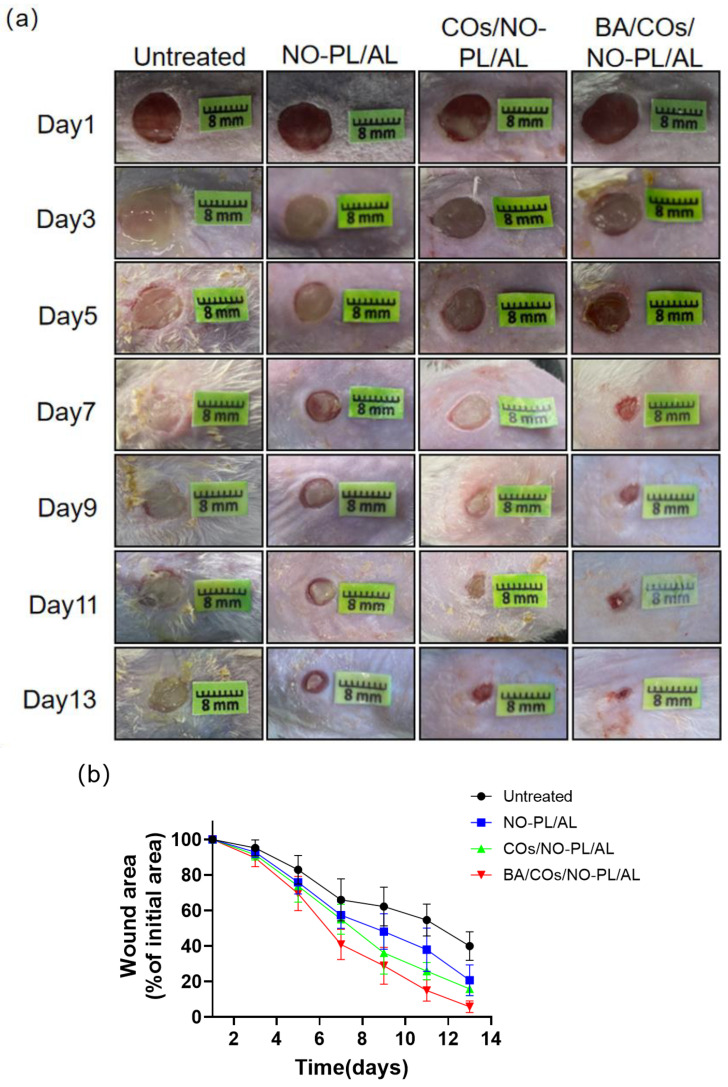

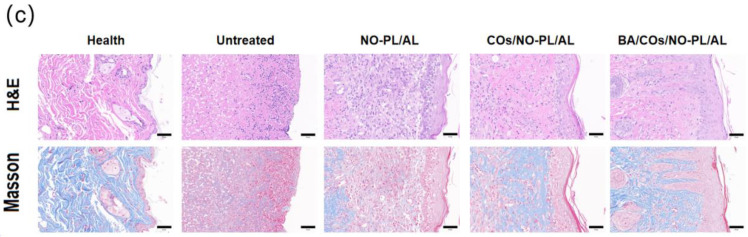
(**a**) Representative photographs of mice treated with NO-PL/AL, COs/NO-PL/AL, and BA/COs/NO-PL/AL healing burn wounds infected with MRPA. (**b**) MRPA-infected wound area reduction rate (%). Data are expressed as mean ± SD (n = 6). (**c**) Histological analysis of MRPA-infected wounds at 14 days post-injury. Bar = 50 μm.

The aforementioned findings indicate that BA/COs/NO-PL/AL gels, which possess favorable physical antimicrobial and anti-inflammatory characteristics, hold great potential as therapeutic agents for wound infections. The results of this investigation, which assessed the therapeutic impact of wound infections through the application of NO-PL/AL, COs/NO-PL/AL, and BA/COs/NO-PL/AL gels, are illustrated in Figure 7. Figure 7a demonstrates a reduction in the trauma area across all groups; however, the impact was particularly significant in the BA/COs/NO-PL/AL group. Mild infection and inflammation were observed on the final day of treatment in the untreated, NO-PL/AL, and COs/NO-PL/AL groups, whereas the wound healing process was nearly complete in the BA/COs/NO-PL/AL group. The measurement of the wound healing process was accomplished through the wound healing rate (Figure 7b), in which the initial area (100 percent) of the lesion was recorded on day one. The trabecular area percentages for untreated, COs/NO-PL/AL, BA/COs/NO-PL/AL, and NO-PL/AL were as follows: 65.94 ± 10.79%, 57.31 ± 6.84%, 55.00 ± 7.15%, and 46.53 ± 9.7% on day 7, and 14 days after the procedure, 20.71 ± 7.91%, 15.82 ± 3.25%, and 5.69 ± 3.05% on day 13, and 20.71 ± 7.91%, 15.82 ± 3.25%, and 5.69 ± 3.05% 13 days after the procedure. These results indicate that while wounds healed in all groups, BA/COs/NO-PL/AL promoted wound healing the most effectively, most likely as a consequence of BA/COs’ anti-inflammatory and antimicrobial properties.

To assess the efficacy of BA/COs/NO-PL/AL gels in promoting wound healing, histological examination of cutaneous wounds was conducted employing H&E and Masson’s trichrome staining techniques (Figure 7c). The H&E staining results indicated that all three groups underwent varying degrees of healing after a 14-day treatment period. Specifically, the BA/COs/NO-PL/AL group exhibited the most favorable repair outcomes characterized by a thinner epidermis and mild hyperplastic scarring, which are characteristics typically associated with healthy skin. Massive quantities of granulocytes and monocytes were observed in the untreated group, the COs/NO-PL/AL group, and the NO-PL/AL group. The results of Masson’s trichrome staining were utilized to distinguish between muscle fiber and collagen fiber conditions in the trauma. The collagen content of these substances can serve as an indicator for assessing the efficacy of wound healing. As one progressed from the untreated group to the BA/COs/NO-PL/AL group, the abundance and intensity of the blue hue increased, suggesting a progressive augmentation in collagen content. The incorporation of BA/COs into NO-PL/AL hydrogels promoted wound healing and significantly enhanced collagen deposition.

### 2.8. Wound Colony Load and Inflammatory Expression

**Figure 8:**
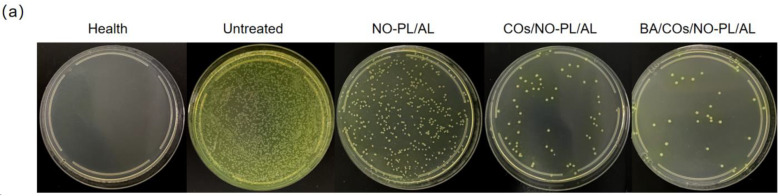

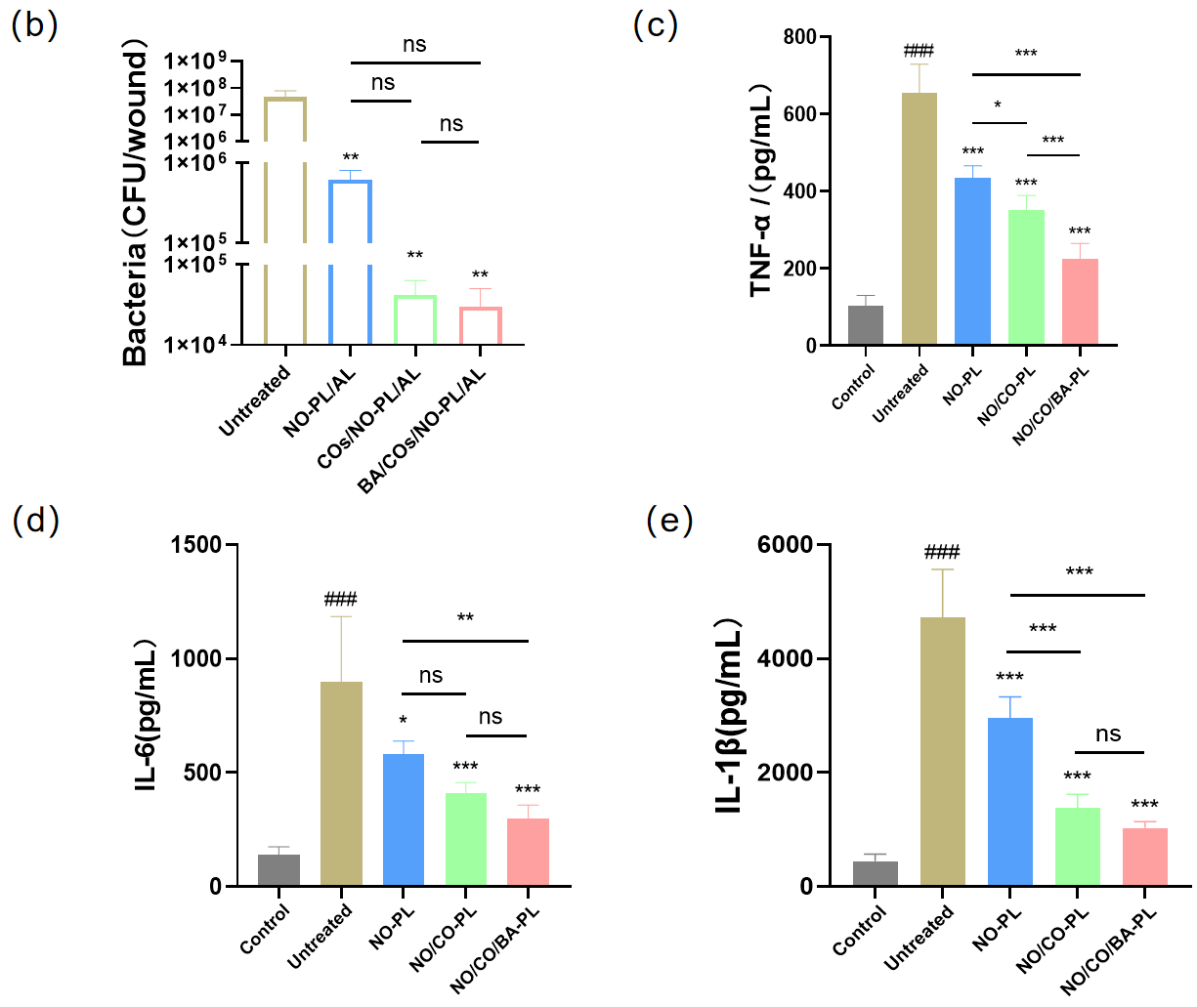
(**a**) Photograph of MRPA colonies obtained from wound tissue. (**b**) Quantitative analysis of bacterial counts. Data are expressed as mean ± SD (n = 3). (**c**) Expression of TNF-α in wound tissue. Data are expressed as mean ± SD (n = 6). (**d**) Expression of IL-6 in wound tissue. Data are expressed as mean ± SD (n = 6). (**e**) Expression of L-1β in wound tissue. Data are expressed as mean ± SD (n = 6). ^###^ *p* < 0.001 vs. control. * *p* < 0.05, ** *p* < 0.01, *** *p* < 0.01 vs. Untreated. ns denotes *p* > 0.05.

The intricate nature of the wound healing process, which is impacted by numerous variables, including bacterial infection and the expression of inflammatory factors, is widely acknowledged [38,39]. In order to assess the therapeutic efficacy of BA/COs/NO-PL/AL gels on MRSA-infected wounds, additional research was conducted to determine how these gels affected MRSA colony loadings and inflammatory factors in wound tissues. The hydrogel treatment resulted in substantially lower MRSA colony loadings in all groups compared to the untreated group (Figure 8a,b). This suggests that the gel exhibited a durable and substantial inhibitory impact on the growth of traumatic MRSA. While hydrogel does facilitate wound healing by generating a moist environment, this moisture also increases the risk of bacterial infections, which have the potential to exacerbate the condition [40]. From this perspective, the sustained antimicrobial activity of BA/COs/NO-PL/AL gels in wounds rendered them an ideal wound dressing.

Furthermore, there is a positive correlation between wound healing and cytokines, which may serve as an indirect indicator of wound healing efficacy [39]. The results presented in Figure 8c,d,e indicate that the concentrations of TNF-α, IL-6, and IL-1β were considerably elevated in the untreated group of mice relative to the control group (*p* < 0.05). IL-1, IL-6, and TNF- were all downregulated in the lesion tissue by the hydrogel groups; however, the gel group containing BA/COs/NO-PL/AL exhibited the most pronounced effect. This result provides additional confirmation that the BA/COs/NO-PL/AL gels inhibit wound bacterial proliferation, reduce wound inflammation, and promote wound healing, indicating that the BA/COs/NO-PL/AL gels may have the capacity to facilitate the repair of infected wounds.

## 3. Conclusions

In this work, we successfully designed and developed a dual-delivery temperature-sensitive hydrogel (BA/COs/NO-PL/AL) with antibacterial and anti-inflammatory BA and nitric oxide for wound healing in MRPA infections. Doping BA/COs submicron emulsion in NO-PL/AL effectively improved the anti-inflammatory activity and wound healing ability of the hydrogel. The hydrogel showed low cytotoxicity against L929 mouse fibroblasts and good anti-inflammatory activity against LPS-induced RAW264.7 cells. BA/COs/NO-PL/AL showed significant antibacterial activity against both MRPA and MRSA. In addition, BA/COs/NO-PL/AL treatment of MRPA-infected wounds promoted wound healing, reduced wound bacterial load, and downregulated the expression of IL-1β, IL-6, and TNF-α in wound tissue. In conclusion, BA/COs/NO-PL/AL hydrogel is expected to be a novel wound dressing material with great potential for promoting wound healing in MRPA infections.

## 4. Materials and Methods

### 4.1. Materials

Sodium alginate (AL) (~200 kDa, the ratio of mannuronate/guluronate at ~1.56) was purchased from Yuanye Biotechnology Ltd. (Shanghai, China). Sodium nitrite was purchased from Macklin Biochemical Technology Ltd. (Shanghai, China). Brevilin A (BA) was purchased from Yongjian Pharmaceutical Science and Technology Co. (Taizhou, China). Camellia oil (CO) was purchased from Daheng (Ceheng counties, China). Pluronic F127 (PL), 2,2,2-tribromoethanol, and tert-pentyl alcohol (2-methyl-2-butanol) (an anesthetic component of avidin) were purchased from Sigma-Aldrich (St. Louis, MO, USA). Glutathione (GSH) was purchased from Wako Pure Chemical (Osaka, Japan). Methicillin-resistant Staphylococcus aureus 3089 (MRSA) and multidrug-resistant Pseudomonas aeruginosa 2200 (MRPA) were purchased from the Korean National Research Resource Bank (KNRRB, Seoul, Republic of Korea). Ctrimida Agar Medium was purchased from Shandong Top Biological Engineering Co. (Qingdao, China). Luria Bertani (LB) medium was purchased from Shanghai Bo Microbiology Technology Co. (Shanghai, China). DMEM medium was purchased from Thermo Scientific (Waltham, MA, USA). fetal bovine serum and trypsin were purchased from Biological Industries (Kibbutz Beit Haemek, Israel). Masson’s trichrome stain (connective tissue stain) was purchased from Servicebio (Wuhan, China). All other reagents and solvents are of analytical grade. The nitric oxide assay Kit was purchased from Beyotime (Shanghai, China). Mouse interleukin 1β (IL-1β), mouse interleukin 6 (IL-6), and tumor necrosis factor-α (TNF-α) enzyme-linked immunosorbent assay kits were purchased from Solarbio Technology Ltd. (Shanghai, China).

### 4.2. BA Submicron Emulsion Preparation

BA submicron emulsions were prepared according to the ultrasonic emulsification method [41]. With some modifications, 9.6 mg of BA was dissolved in 2.4 g of CO to form an oil phase, and 0.75 g of sucrose ester SE-15 was dissolved with 0.25 g of PEG-400 in 21.6 g of water to form an aqueous phase. Colostrum was prepared through the dropwise addition of the oil phase to the aqueous phase under slow stirring. The prepared colostrum was homogenized through ultrasound-assisted homogenization in a cold water bath (ultrasound conditions: power 350 W, ultrasound time 30 s, gap 10 s, and ultrasound for 6 min), i.e., BA submicron emulsion free COs and BA submicron emulsion containing BA/COs were obtained.

### 4.3. GSNO Synthesis

The NO donor GSNO was synthesized through the reaction of GSH with sodium nitrite (NaNO_2_), as previously described [35]. Briefly, GSH and NaNO_2_ were synthesized by reacting GSH and NaNO_2_ in aqueous hydrochloric acid solution in an ice bath for 40 min under light-avoidance conditions. The final concentration of GSH, NaNO_2_, and HCl was 0.625 M. After precipitation with acetone, the precipitate was collected through vacuum filtration, and after adding cold water to disperse the sediment, it was washed twice with 100% acetone and three times with ether and freeze-dried. The solid GSNO was stored in a −20 °C refrigerator for subsequent experiments.

### 4.4. Preparation of BA/COs/NO-PL/AL Gels

GSNO-PL/AL hydrogel was prepared as previously reported with minor modifications [35]. First, AL was dissolved into distilled water at room temperature with magnetic stirring. Next, PL was added to the AL solution overnight at 4 °C to completely dissolve the PL powder to obtain PL/AL hydrogel. The GSNO powder was then added to the PL/AL hydrogel, which was subjected to weak magnetic stirring and light protection at 4 °C to form the NO-PL/AL hydrogel. Finally, water, COs, and BA/COs were slowly added to the NO-PL/AL hydrogel in a 1:1 ratio, which was treated with weak magnetic stirring and protection from light at 4 °C. The final products were NO-PL/AL, COs/NO-PL/AL, and BA/COs/NO-PL/AL where the concentrations of PL, AL, and GSNO in the hydrogels were 20% *w*/*v*, 1% *w*/*v*, and 2% *w*/*v*, respectively, which were stored in a 4 °C refrigerator for subsequent experiments. The condition of use is to avoid light and remove from the refrigerator at 4 °C for immediate use.

### 4.5. Particle Size, PDI, and Zeta Potential Measurements

Particle size, polydispersity index (PDI), and zeta potential of COs and BA/COs were determined through the dynamic light scattering (DLS) technique (N4plus Delsa 440SX, Beckman Coulter, London, UK) [42].

### 4.6. Centrifugal Stability Coefficient Ke Value

We took 1 mL of COs and BA/COs solution in a 2 mL centrifuge tube, centrifuged at 4000 rpm/min for 15 min, and then took 20 µL of the lower solution in a 10 mL volumetric flask and mixed it with distilled water. The absorbance A (n = 3) was determined through UV-Vis spectrophotometer at 245 nm using distilled water as a blank control. The absorbance A_0_ of uncentrifuged COs and BA/COs was measured in the same way, and the centrifugal stability parameter Ke was calculated. Smaller Ke indicates a more stable emulsion [7]. The formula is as follows:

(1) $$Ke=\frac{A_{0}-A}{A_{0}}$$

### 4.7. Thermal Responsiveness of Hydrogel

The dynamic viscosities of PL/AL, NO-PL/AL, COs/NO-PL/AL, and BA/COs/NO-PL/AL were determined using an ETT CP5000 Lamyett rheometer with a shear rate of 10 S^−1^. The inflection point of the hydrogel formulation was determined from the dynamic viscosity–temperature graph through a programmed temperature increase at a rate of 5 °C/min in the range of 5–50 °C.

### 4.8. Rheology of Hydrogels

The rheological properties of PL/AL, NO-PL/AL, COs/NO-PL/AL, and BA/COs/NO-PL/AL gels were investigated as a function of temperature using a hybrid rheometer (specification: DHR^−1^, TA Instruments Waters, Newcastle, DE, USA). Rheological studies were carried out on 40 mm parallel plates in the temperature range of 5–50 °C with a heating rate of 1 °C/min [43].

### 4.9. Thermal Analysis of Hydrogels

The thermal properties of the hydrogels were determined through the differential scanning calorimetry (DSC) technique (METTLER TOLEDO, Zurich, Switzerland) using a DSC Q200 device. In total, 5 mg of hydrogel samples was analyzed in the range of 2–50 °C with a heating rate of 1 °C/min, and the gas chamber was purged with nitrogen (50 mL/min).

### 4.10. Drug Dissolution

An in vitro kinetic study of diffusion of intact NO and BA from hydrogels was performed using a 12 mL vertical Franz diffusion cell (LOGAN DSC-800, Shanghai, China; founded in Somerset, NJ, USA) [44,45]. The cell consists of a donor and acceptor chamber separated by a hydrophilic nitrocellulose membrane with a porosity of 20 nm and a diameter of 25 mm (Pall Corporation, Port Washington, NY, USA). The donor chamber was filled with 2 g of hydrogel (NO-PL/AL, CO/NO-PL/AL, and BA/CO/NO-PL/AL). At 32 °C, 12 mL of PBS containing 30% ethanol was added and stirred with a miniature paddle at 300 rpm, and 1.2 mL was removed from the receptor vesicles at various time intervals (0.5, 1, 1, 2, 3, 4, 5, 6, 12, 24, 36, and 48 h) and replaced with an equal amount of PBS containing 30% ethanol. To quantify NO in the hydrogel, NO content was determined according to the Griess kit instructions [46]. The BA content in the receiving solution was determined through high-performance liquid chromatography according to the method of Chinese Pharmacopoeia. The chromatographic conditions were Agilent C18, 4.6 × 250 mm, 5 μm; the mobile phase was acetonitrile/water (45:55, *v*/*v*); the detection wavelength was 225 nm; the flow rate was 1 mL/min; the column temperature was 25 °C; and the injection volume was 10 μL.

### 4.11. Cytotoxic Activity of Hydrogels against L929

Cytotoxicity of NO-PL/AL, COs/NO-PL/AL, and BA/COs/NO-PL/AL hydrogels on L929 mouse fibroblasts was evaluated using the tetramethyl azole salt colorimetric method (MTT) [35]. L929 cells were inoculated into 96-well plates at 6 × 10^3^ per well, incubated for 24 h, replaced with fresh culture medium containing hydrogels of different concentrations (2, 10, 20, 100, 200 mg/mL), and incubated at 37 °C for 24 h. After that, 10 µL of MTT at 5 mg/mL was added to each well and incubated for 4 h at 37 °C; the supernatant was discarded, 160 µL of DMSO was added and incubated for 10 min while protected from light, and the absorbance was measured using an enzyme marker at 490 nm. Data are expressed as mean ± standard deviation (SD) of six replicates. Cell survival was calculated using the following formula:

Cell viability (%) = (OD_processed cell_)/(OD_control cell_) × 100% (2)

### 4.12. RAW 264.7 Determination of Cell Viability

A total of 100 µL/well of RAW264.7 cells (2.5 × 10^5^ cells) was inoculated into 96-well plates and incubated for 24 h. Then, 70 µL of supernatant was aspirated and discarded, and 60 µL of fresh medium was added followed by 10 µL of samples (BA, COs, NO-PL/AL, COs/NO-PL/AL, and BA/COs/NO-PL/AL). After 24 h of incubation, an MTT assay was performed [47].

### 4.13. In Vitro Anti-Inflammatory Activity

RAW264.7 cells (2.5 × 10^5^ cells) were inoculated in 96-well plates and incubated for 24 h before changing the solution. After adding 10 µL of LPS for 2 h, samples (BA, COs, NO-PL/AL, COs/NO-PL/AL, and BA/COs/NO-PL/AL) were added and incubated for 24 h. The cell supernatants were taken to determine the levels of TNF-α and IL-6 using an enzyme-linked immunosorbent assay kit [47].

### 4.14. In Vitro Antibacterial Activity

Antimicrobial activity of hydrogels evaluated using MRSA and MRPA [48]. Briefly, the hydrogel sample (600 µL) was added to a 24-well plate and mixed with 1.8 mL of bacterial suspension at a final concentration of 1 × 10^8^ CFU/mL and co-cultured at 37 °C for 24 h. After serial dilution of the mixture, 100 µL of the homogenate was applied to a bacterial solid culture plate and cultured at constant temperature and humidity (temperature: 37 °C; humidity: 60%) in an incubator for 24 h. The bacteria in the medium were photographed, and the antibacterial rate (AR) was calculated. The antimicrobial rate of the hydrogel was calculated using the following equation:

(3) $$AR=\frac{N_{PL/AL}-N_{S}}{N_{PL/AL}}\times 100\%$$

where N_PL/AL_ is the number of colonies in PL/AL hydrogels and N_S_ is the number of colonies in hydrogels in NO-PL/AL, COs/NO-PL/AL, and BA/COs/NO-PL/AL.

### 4.15. In Vivo Evaluation of Wound Healing

#### 4.15.1. Healing Effect of Hydrogel on MRPA-Infected Wounds

All animal experiments were reviewed and approved by the Animal Protection and Use Committee of Guizhou Medical University on 25 September 2023 (Use Permit: SYXK (Gui) 2023-0002). ICR mice (7–8 weeks old), body mass (30 ± 2) g, were purchased from Beijing Chemical Fukang Science and Technology Co., Ltd., license SCXK (Beijing, China) 2019-0008. Animals were housed in the Animal Centre of Guizhou Medical University with a 12 h light/12 h dark cycle at room temperature.

MRPA-infected traumatized mice were prepared according to pre-methods [37]. This model was used to study the effectiveness of BA/COs/NO-PL/AL hydrogel in promoting wound healing. The mice were anesthetized, and the hair on their backs was removed using an electric razor and depilatory cream. A circular full skin wound of 8 mm in diameter was then made on the back using a perforator. Infection was induced by inoculating each wound with 20 µL of MRPA suspension (6 × 10^9^ CFU/mL). Mice were randomly divided into untreated groups, NO-PL/AL, COs/NO-PL/AL, and BA/COs/NO-PL/AL hydrogels. After photographing the wound every other day after the injury, 20 µL of hydrogel was applied to the wound and covered with 3M™ Tegaderm™ film and then fixed with gauze and tape for 14 d. The wound area was measured using ImageJ software (1.8.0_127), and calculation of the wound reduction rate occurred using the following equation:

Wound size reduction (%) = W_t_/W_0_ × 100 (4)

where W_0_ is the wound area on the day of surgery and W_t_ is the wound area at the specified time.

#### 4.15.2. Wound Colony Load

On the last day of the experiment, the mice were anesthetized and executed through cervical dislocation; the skin tissues around the wounds were quickly collected, and the tissues were weighed, ground, and diluted with sterile saline to make a 1:10 sample homogenate. Then, 1–3 sample homogenates of suitable dilution were selected and applied on the bacterial solid culture plate and incubated in the incubator for 24 h. Then, bacteria on the medium were photographed, and colony loading per unit weight of the tissues was calculated. In total, 3 parallel samples were collected in each group.

#### 4.15.3. Detection of Inflammatory Biomarkers in Skin Wound Tissue

Weighed skin tissues were homogenized 1:9 with pre-cooled PBS (0.01 M, pH = 7.4) solution. Finally, the homogenate was centrifuged at 4 °C for 10 min at 5000× *g*. Concentrations of IL-1β, IL-6, and TNF-α were identified using enzyme-linked immunosorbent assay kits according to the manufacturer’s protocol.

#### 4.15.4. Organizational Assessment

Wound tissue was collected from each group on day 14 and fixed with 10% paraformaldehyde, embedded, and cut longitudinally into 4 µm thick sections. To evaluate the cellular morphology and collagen formation of the regenerated skin tissue, HE staining and Masson staining were performed. The sections were imaged using a light microscope.

### 4.16. Statistical Analysis

Data are expressed as mean ± standard deviation (SD). Statistical analyses were performed using GraphPad Prism 8.0 software, and one-way ANOVA and Tukey’s HSD test were used for comparisons between groups.

## Figures and Tables

**Table 1:** Characterization of submicron emulsion.

Name	Size (nm)	PDI	Zeta Potential (mV)	Ke
COs	237.82 ± 0.54	0.183 ± 0.04	−38.67 ± 0.19	0.13 ± 0.09
BA/COs	239.18 ± 1.47	0.183 ± 0.04	−38.25 ± 0.27	0.22 ± 0.09

Data are expressed as mean ± SD (n = 3).

**Table 2:** Characterization of the hydrogels.

Hydrogels	T_onset_ (°C)	T_peak_ (°C)	T_endset_ (°C)	ΔH (mJ)	T_gel_ (°C)	Loading (%)
PL/AL	17.45	19.82	25.55	−83.21	24.7	N.D.
NO-PL/AL	15.07	17.87	21.97	−84.54	22.4	0.172 ± 0.01% *
COs/NO-PL/AL	13.45	14.72	20.58	−87.49	21.9	0.169 ± 0.01% *
BA/COs/NO-PL/AL	13.52	14.65	20.52	−77.78	21.8	0.171 ± 0.02% * 0.009 ± 0.00% ^#^

N.D. is no data; * is NO loading; ^#^ is BA loading. Data are expressed as mean ± SD (n = 3).

## Data Availability

The data presented in this study are openly available in article.
